# 2-{4-[(1,3-Benzodioxol-5-yl)meth­yl]piperazin-1-yl}pyrimidine

**DOI:** 10.1107/S1600536813016851

**Published:** 2013-06-22

**Authors:** Chunli Wu, Jieming Li, Huijie Wei, Ye Hang, Yueming Jiang

**Affiliations:** a School of Pharmaceutical Sciences, Zhengzhou University, Zhengzhou 450001, People’s Republic of China

## Abstract

In the title compound, C_16_H_18_N_4_O_2_, known also as peribedil, the dihedral angle between the mean planes of the pyrimidine and benzene rings is 56.5 (8)°. The 1,3-dioxole fragment adopts an envelope conformation with the methyl­ene C atom forming the flap; this atom deviates by 0.232 (3) Å from the plane defined by the remaining atoms of the 1,3-benzodioxole unit. In the crystal, C—H⋯π inter­actions between *c*-glide-related mol­ecules arrange them into columns extending along the *c-*axis direction. The columns related by a unit translation along the *b* axis are packed into (100) layers *via* another C—H⋯π inter­action involving the pyrimidine ring as an acceptor.

## Related literature

For details of the synthesis of piribedil, see: Duncton *et al.* (2006 ▶); Conroy & Denton (1953 ▶); Hamid *et al.* (2007 ▶). For the pharmacological activity of the title compound, see: Rondot *et al.* (1992 ▶).
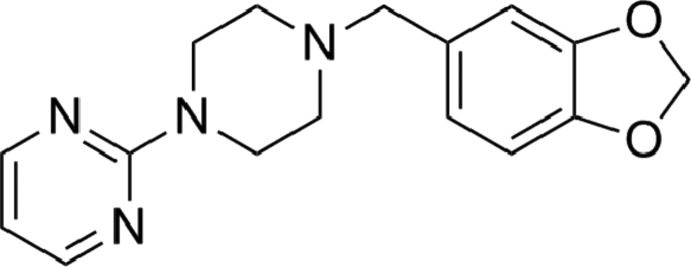


## Experimental

### 

#### Crystal data

C_16_H_18_N_4_O_2_
*M* *_r_* = 298.34
Orthorhombic, 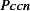

*a* = 21.3085 (6) Å
*b* = 18.6249 (4) Å
*c* = 7.48851 (19) Å
*V* = 2971.95 (12) Å^3^
*Z* = 8
Cu *K*α radiation
μ = 0.74 mm^−1^
*T* = 291 K
0.25 × 0.2 × 0.2 mm

#### Data collection

Agilent Xcalibur (Eos, Gemini) diffractometer
Absorption correction: multi-scan (*CrysAlis PRO*; Agilent, 2012 ▶)’ *T*_min_ = 0.910, *T*_max_ = 1.000
6334 measured reflections
2635 independent reflections
2186 reflections with *I* > 2σ(*I*)
*R*_int_ = 0.019

#### Refinement

*R*[*F* ^2^ > 2σ(*F* ^2^)] = 0.039
*wR*(*F* ^2^) = 0.112
*S* = 1.03
2635 reflections
200 parameters
H-atom parameters constrained
Δρ_max_ = 0.13 e Å^−3^
Δρ_min_ = −0.14 e Å^−3^

### 

Data collection: *CrysAlis PRO* (Agilent, 2012 ▶); cell refinement: *CrysAlis PRO*; data reduction: *CrysAlis PRO*; program(s) used to solve structure: *SHELXS97* (Sheldrick, 2008 ▶); program(s) used to refine structure: *SHELXL97* (Sheldrick, 2008 ▶); molecular graphics: *OLEX2* (Dolomanov *et al.*, 2009 ▶); software used to prepare material for publication: *OLEX2*.

## Supplementary Material

Crystal structure: contains datablock(s) I, global. DOI: 10.1107/S1600536813016851/gk2582sup1.cif

Structure factors: contains datablock(s) I. DOI: 10.1107/S1600536813016851/gk2582Isup2.hkl

Supplementary material file. DOI: 10.1107/S1600536813016851/gk2582Isup3.cml

Additional supplementary materials:  crystallographic information; 3D view; checkCIF report

## Figures and Tables

**Table 1:** Hydrogen-bond geometry (Å, °) *Cg*1 is the centroid of the pyrimidine ring and *Cg*2 is the centroid of the benzene ring.

*D*—H⋯*A*	*D*—H	H⋯*A*	*D*⋯*A*	*D*—H⋯*A*
C2—H2⋯*Cg*1^i^	0.93	2.83	3.6771 (17)	152
C9—H9*B*⋯*Cg*1^ii^	0.97	2.92	3.8090 (18)	152
C16—H16*A*⋯*Cg*2^iii^	0.97	2.80	3.689 (2)	153

Symmetry codes: (i) 
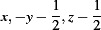
; (ii) 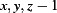; (iii) 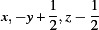.

**Fig. 1.**
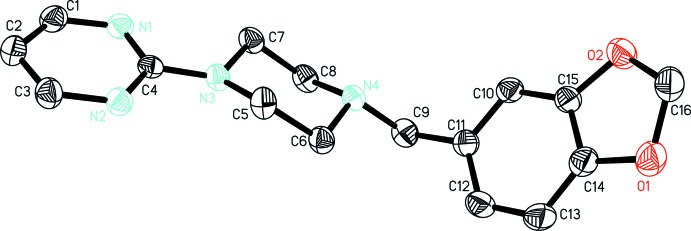
The molecular structure of the compound, with 30% probability displacement ellipsoids for non-hydrogen atoms.

**Fig. 2.**
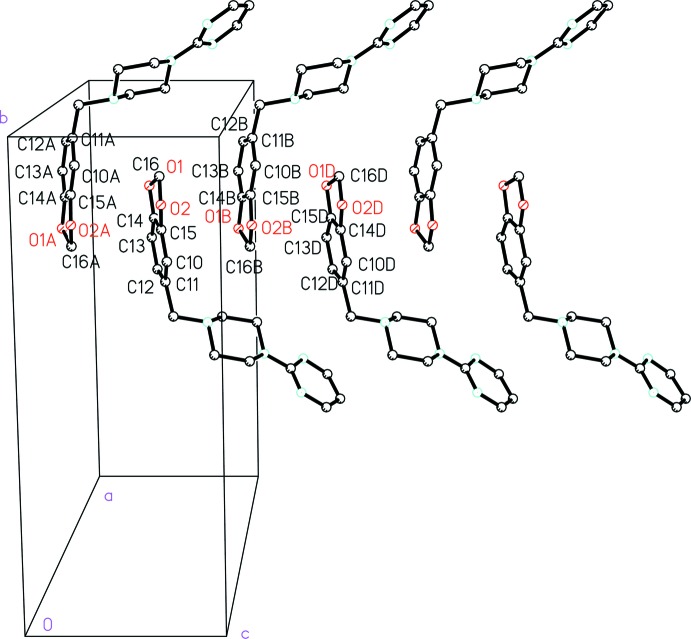
A view of the column along the c axis formed via C—H···π stacking interactions (symmetry code: A x,1.5 - y,z - 0.5; B x,1.5 - y,z + 0.5; D x,y,1 + z.).

## supplementary crystallographic information

### Comment

Experimental investigation shows that the dopamine agonist, piribedil, is active in the treatment of Parkinson's disease, particularly with regard to tremor (Rondot *et al.*, 1992). We report herein the synthesis (Hamid *et al.*, 2007) and the crystal structure of the title compound. The benzene and pyrimidine rings subtend a dihedral angle of 56.5 (8)°. The benzo[1,3]dioxole fragments, the dihedral angle between O1—C16—O2 plane and the remaining 8 atoms of the bicyclic fragment (O1—C14—C13—C12—C11—C10—C15—O2) is 16.1 (1)°. Piperazine fragments, the dihedral angle between C5—C8 plane and C5—N3—C7 plane is 29.7 (3) °, C5—C8 plane and C6—N4—C8 plane is 23.9 (7) °. In the crystal, the molecules associate through C—H···π interactions (see table 1).

### Experimental

The triethylamine catalyzed reaction of 5-chlorobenzo[*d*][1,3]dioxole (8.5 mmol) and 2-(piperazin-1-yl)pyrimidine (8.1 mmol) was carried out in isopropyl alcohol (10 mL). The reaction mixture was refluxed for 2 h to afford the title compound. Colorless blocks of the title compound were obtained by recrystallization from ethanol. Crystals suitable for X-ray analysis were grown from methyl alcohol-ethyl acetate solution at room temperature by slow evaporation over two weeks.

### Refinement

H atoms were placed in calculated positions and allowed to ride on their carriers with C—H distances 0.93–0.97 Å and *U*_iso_(H)=1.2*U*_eq_(C).

### Crystal data

**Table:**

C_16_H_18_N_4_O_2_	*D*_x_ = 1.334 Mg m^−^^3^
*M**_r_* = 298.34	Melting point = 370–372 K
Orthorhombic, *P**c**c**n*	Cu *K*α radiation, λ = 1.5418 Å
*a* = 21.3085 (6) Å	Cell parameters from 2402 reflections
*b* = 18.6249 (4) Å	θ = 3.2–67.0°
*c* = 7.48851 (19) Å	µ = 0.74 mm^−^^1^
*V* = 2971.95 (12) Å^3^	*T* = 291 K
*Z* = 8	Block, colorless
*F*(000) = 1264	0.25 × 0.2 × 0.2 mm

### Data collection

**Table:**

Agilent Xcalibur (Eos, Gemini) diffractometer	2635 independent reflections
Radiation source: Enhance (Cu) X-ray Source	2186 reflections with *I* > 2σ(*I*)
Graphite monochromator	*R*_int_ = 0.019
Detector resolution: 16.2312 pixels mm^-1^	θ_max_ = 67.1°, θ_min_ = 3.2°
ω scans	*h* = −25→24
Absorption correction: multi-scan (*CrysAlis PRO*; Agilent, 2012)'	*k* = −22→19
*T*_min_ = 0.910, *T*_max_ = 1.000	*l* = −7→8
6334 measured reflections	

### Refinement

**Table:**

Refinement on *F*^2^	Secondary atom site location: difference Fourier map
Least-squares matrix: full	Hydrogen site location: inferred from neighbouring sites
*R*[*F*^2^ > 2σ(*F*^2^)] = 0.039	H-atom parameters constrained
*wR*(*F*^2^) = 0.112	*w* = 1/[σ^2^(*F*_o_^2^) + (0.0574*P*)^2^ + 0.4246*P*] where *P* = (*F*_o_^2^ + 2*F*_c_^2^)/3
*S* = 1.03	(Δ/σ)_max_ < 0.001
2635 reflections	Δρ_max_ = 0.13 e Å^−^^3^
200 parameters	Δρ_min_ = −0.14 e Å^−^^3^
0 restraints	Extinction correction: *SHELXL*, Fc^*^=kFc[1+0.001xFc^2^λ^3^/sin(2θ)]^-1/4^
Primary atom site location: structure-invariant direct methods	Extinction coefficient: 0.00143 (18)

### Special details

**Table:**

Experimental. ^1^H NMR (400 MHz, CDCl_3_, p.p.m.): 8.30 (*d*, *J* = 4.7 Hz, 1H), 6.90 (*s*, 1H), 6.76 (*s*, 1H), 6.47 (*s*, *J* = 4.7 Hz, 1H), 6.20 (*dt*, *J* = 10.6, 2.2 Hz, 1H), 5.95 (*s*, 1H), 3.89–3.66 (*m*, 2H), 3.46 (*s*, 1H), 2.58–2.27 (*m*, 2H); ^13^C NMR (101 MHz, CDCl_3_, p.p.m.): 161.67, 157.70, 147.68, 146.66, 131.91, 122.23 ,109.72, 109.50, 107.90, 100.91, 62.89, 52.85, 43.69; ESI–HRMS *m*/*z*: 299.1506 (calculated for C_16_H_19_N_4_O_2_ [*M* + 1]^+^: 299.1508).
Geometry. All e.s.d.'s (except the e.s.d. in the dihedral angle between two l.s. planes) are estimated using the full covariance matrix. The cell e.s.d.'s are taken into account individually in the estimation of e.s.d.'s in distances, angles and torsion angles; correlations between e.s.d.'s in cell parameters are only used when they are defined by crystal symmetry. An approximate (isotropic) treatment of cell e.s.d.'s is used for estimating e.s.d.'s involving l.s. planes.
Refinement. Refinement of *F*^2^ against ALL reflections. The weighted *R*-factor *wR* and goodness of fit *S* are based on *F*^2^, conventional *R*-factors *R* are based on *F*, with *F* set to zero for negative *F*^2^. The threshold expression of *F*^2^ > σ(*F*^2^) is used only for calculating *R*-factors(gt) *etc*. and is not relevant to the choice of reflections for refinement. *R*-factors based on *F*^2^ are statistically about twice as large as those based on *F*, and *R*- factors based on ALL data will be even larger.

### Fractional atomic coordinates and isotropic or equivalent isotropic displacement parameters (Å^2^)

**Table:**

	*x*	*y*	*z*	*U*_iso_*/*U*_eq_	
O1	0.65086 (6)	0.79907 (7)	0.4624 (2)	0.0734 (4)	
O2	0.55046 (6)	0.77275 (6)	0.55466 (19)	0.0663 (4)	
N1	0.59429 (6)	0.33262 (7)	1.31759 (19)	0.0498 (3)	
N2	0.69678 (6)	0.38529 (7)	1.31927 (17)	0.0476 (3)	
N3	0.62385 (6)	0.41598 (6)	1.10454 (18)	0.0457 (3)	
N4	0.59232 (6)	0.49704 (6)	0.79397 (17)	0.0440 (3)	
C1	0.61119 (9)	0.29307 (9)	1.4583 (2)	0.0557 (4)	
H1	0.5819	0.2616	1.5070	0.067*	
C2	0.66955 (9)	0.29663 (9)	1.5342 (2)	0.0575 (4)	
H2	0.6805	0.2685	1.6318	0.069*	
C3	0.71099 (9)	0.34407 (9)	1.4582 (2)	0.0545 (4)	
H3	0.7511	0.3475	1.5066	0.065*	
C4	0.63848 (7)	0.37709 (7)	1.2534 (2)	0.0404 (3)	
C5	0.65864 (8)	0.48112 (8)	1.0596 (2)	0.0508 (4)	
H5A	0.7019	0.4762	1.0982	0.061*	
H5B	0.6404	0.5218	1.1219	0.061*	
C6	0.65682 (7)	0.49460 (8)	0.8609 (2)	0.0489 (4)	
H6A	0.6775	0.5398	0.8348	0.059*	
H6B	0.6796	0.4568	0.7998	0.059*	
C7	0.56029 (8)	0.41465 (9)	1.0326 (2)	0.0503 (4)	
H7A	0.5350	0.4509	1.0914	0.060*	
H7B	0.5414	0.3682	1.0552	0.060*	
C8	0.56174 (8)	0.42877 (8)	0.8347 (2)	0.0512 (4)	
H8A	0.5841	0.3902	0.7755	0.061*	
H8B	0.5192	0.4295	0.7890	0.061*	
C9	0.59173 (9)	0.51044 (9)	0.6002 (2)	0.0545 (4)	
H9A	0.5501	0.5006	0.5541	0.065*	
H9B	0.6208	0.4777	0.5426	0.065*	
C10	0.56549 (8)	0.64162 (9)	0.5773 (2)	0.0495 (4)	
H10	0.5247	0.6319	0.6144	0.059*	
C11	0.60958 (8)	0.58649 (9)	0.5536 (2)	0.0491 (4)	
C12	0.66915 (9)	0.60274 (11)	0.4929 (3)	0.0619 (5)	
H12	0.6975	0.5655	0.4746	0.074*	
C13	0.68819 (9)	0.67322 (11)	0.4582 (3)	0.0691 (5)	
H13	0.7284	0.6837	0.4175	0.083*	
C14	0.64478 (8)	0.72593 (10)	0.4871 (2)	0.0567 (4)	
C15	0.58497 (8)	0.71057 (9)	0.5435 (2)	0.0494 (4)	
C16	0.59495 (9)	0.82922 (10)	0.5360 (3)	0.0644 (5)	
H16A	0.6037	0.8507	0.6513	0.077*	
H16B	0.5786	0.8662	0.4574	0.077*	

### Atomic displacement parameters (Å^2^)

**Table:**

	*U*^11^	*U*^22^	*U*^33^	*U*^12^	*U*^13^	*U*^23^
O1	0.0539 (8)	0.0690 (8)	0.0972 (11)	−0.0100 (7)	−0.0008 (7)	0.0219 (8)
O2	0.0523 (7)	0.0565 (7)	0.0901 (9)	0.0039 (6)	0.0045 (7)	0.0059 (6)
N1	0.0442 (7)	0.0486 (7)	0.0564 (8)	−0.0013 (6)	0.0050 (6)	0.0033 (6)
N2	0.0433 (7)	0.0517 (7)	0.0477 (7)	−0.0018 (6)	−0.0037 (6)	0.0038 (6)
N3	0.0380 (7)	0.0450 (6)	0.0542 (7)	−0.0030 (5)	−0.0059 (6)	0.0058 (6)
N4	0.0415 (7)	0.0422 (6)	0.0484 (7)	0.0022 (5)	−0.0063 (5)	−0.0005 (5)
C1	0.0596 (11)	0.0488 (8)	0.0588 (10)	−0.0012 (8)	0.0133 (8)	0.0074 (8)
C2	0.0655 (11)	0.0567 (9)	0.0502 (9)	0.0051 (9)	0.0014 (8)	0.0102 (8)
C3	0.0535 (10)	0.0591 (9)	0.0510 (9)	0.0018 (8)	−0.0078 (8)	0.0040 (8)
C4	0.0403 (7)	0.0372 (7)	0.0439 (8)	0.0042 (6)	0.0034 (6)	−0.0050 (6)
C5	0.0496 (9)	0.0459 (8)	0.0569 (9)	−0.0080 (7)	−0.0131 (7)	0.0060 (7)
C6	0.0406 (8)	0.0501 (8)	0.0560 (9)	−0.0019 (7)	−0.0057 (7)	0.0064 (7)
C7	0.0366 (8)	0.0486 (8)	0.0656 (10)	−0.0008 (7)	−0.0031 (7)	0.0062 (8)
C8	0.0418 (8)	0.0476 (8)	0.0642 (10)	−0.0012 (7)	−0.0128 (7)	−0.0025 (8)
C9	0.0568 (10)	0.0563 (9)	0.0505 (9)	0.0028 (8)	−0.0106 (8)	−0.0043 (8)
C10	0.0375 (8)	0.0616 (9)	0.0495 (9)	−0.0008 (7)	−0.0004 (7)	0.0046 (7)
C11	0.0461 (9)	0.0599 (9)	0.0412 (8)	0.0034 (7)	−0.0069 (6)	0.0020 (7)
C12	0.0467 (10)	0.0742 (11)	0.0649 (11)	0.0142 (9)	0.0008 (8)	0.0053 (9)
C13	0.0371 (9)	0.0875 (13)	0.0827 (13)	0.0006 (9)	0.0073 (9)	0.0176 (11)
C14	0.0433 (9)	0.0685 (10)	0.0582 (9)	−0.0046 (8)	−0.0038 (7)	0.0129 (8)
C15	0.0411 (8)	0.0580 (9)	0.0490 (8)	0.0055 (7)	−0.0030 (7)	0.0055 (7)
C16	0.0618 (12)	0.0592 (10)	0.0723 (12)	−0.0022 (9)	−0.0057 (9)	0.0125 (9)

### Geometric parameters (Å, º)

**Table:**

O1—C14	1.381 (2)	C6—H6A	0.9700
O1—C16	1.428 (2)	C6—H6B	0.9700
O2—C15	1.374 (2)	C7—H7A	0.9700
O2—C16	1.423 (2)	C7—H7B	0.9700
N1—C1	1.335 (2)	C7—C8	1.505 (2)
N1—C4	1.343 (2)	C8—H8A	0.9700
N2—C3	1.328 (2)	C8—H8B	0.9700
N2—C4	1.345 (2)	C9—H9A	0.9700
N3—C4	1.365 (2)	C9—H9B	0.9700
N3—C5	1.4611 (19)	C9—C11	1.508 (2)
N3—C7	1.458 (2)	C10—H10	0.9300
N4—C6	1.4636 (19)	C10—C11	1.403 (2)
N4—C8	1.4610 (19)	C10—C15	1.373 (2)
N4—C9	1.472 (2)	C11—C12	1.382 (3)
C1—H1	0.9300	C12—H12	0.9300
C1—C2	1.369 (3)	C12—C13	1.398 (3)
C2—H2	0.9300	C13—H13	0.9300
C2—C3	1.373 (3)	C13—C14	1.366 (3)
C3—H3	0.9300	C14—C15	1.373 (2)
C5—H5A	0.9700	C16—H16A	0.9700
C5—H5B	0.9700	C16—H16B	0.9700
C5—C6	1.510 (2)		
			
C14—O1—C16	104.96 (14)	C8—C7—H7A	109.7
C15—O2—C16	105.09 (14)	C8—C7—H7B	109.7
C1—N1—C4	115.70 (15)	N4—C8—C7	111.53 (13)
C3—N2—C4	115.61 (14)	N4—C8—H8A	109.3
C4—N3—C5	120.85 (13)	N4—C8—H8B	109.3
C4—N3—C7	120.32 (13)	C7—C8—H8A	109.3
C7—N3—C5	113.60 (12)	C7—C8—H8B	109.3
C6—N4—C9	110.51 (13)	H8A—C8—H8B	108.0
C8—N4—C6	108.67 (11)	N4—C9—H9A	109.1
C8—N4—C9	110.47 (13)	N4—C9—H9B	109.1
N1—C1—H1	118.5	N4—C9—C11	112.68 (13)
N1—C1—C2	123.09 (16)	H9A—C9—H9B	107.8
C2—C1—H1	118.5	C11—C9—H9A	109.1
C1—C2—H2	121.8	C11—C9—H9B	109.1
C1—C2—C3	116.33 (16)	C11—C10—H10	121.3
C3—C2—H2	121.8	C15—C10—H10	121.3
N2—C3—C2	123.37 (17)	C15—C10—C11	117.31 (15)
N2—C3—H3	118.3	C10—C11—C9	119.30 (15)
C2—C3—H3	118.3	C12—C11—C9	120.90 (16)
N1—C4—N2	125.88 (14)	C12—C11—C10	119.77 (16)
N1—C4—N3	117.34 (14)	C11—C12—H12	118.9
N2—C4—N3	116.75 (13)	C11—C12—C13	122.22 (17)
N3—C5—H5A	109.5	C13—C12—H12	118.9
N3—C5—H5B	109.5	C12—C13—H13	121.7
N3—C5—C6	110.62 (13)	C14—C13—C12	116.67 (17)
H5A—C5—H5B	108.1	C14—C13—H13	121.7
C6—C5—H5A	109.5	C13—C14—O1	128.63 (17)
C6—C5—H5B	109.5	C13—C14—C15	121.84 (17)
N4—C6—C5	111.51 (13)	C15—C14—O1	109.50 (16)
N4—C6—H6A	109.3	C10—C15—O2	127.97 (15)
N4—C6—H6B	109.3	C14—C15—O2	109.87 (15)
C5—C6—H6A	109.3	C14—C15—C10	122.15 (16)
C5—C6—H6B	109.3	O1—C16—H16A	110.2
H6A—C6—H6B	108.0	O1—C16—H16B	110.2
N3—C7—H7A	109.7	O2—C16—O1	107.65 (15)
N3—C7—H7B	109.7	O2—C16—H16A	110.2
N3—C7—C8	109.99 (14)	O2—C16—H16B	110.2
H7A—C7—H7B	108.2	H16A—C16—H16B	108.5
			
O1—C14—C15—O2	0.8 (2)	C7—N3—C5—C6	−52.15 (18)
O1—C14—C15—C10	179.46 (16)	C8—N4—C6—C5	−58.80 (17)
N1—C1—C2—C3	0.3 (3)	C8—N4—C9—C11	167.44 (13)
N3—C5—C6—N4	54.89 (18)	C9—N4—C6—C5	179.83 (13)
N3—C7—C8—N4	−56.91 (17)	C9—N4—C8—C7	−178.59 (14)
N4—C9—C11—C10	−76.79 (19)	C9—C11—C12—C13	−176.31 (18)
N4—C9—C11—C12	101.32 (19)	C10—C11—C12—C13	1.8 (3)
C1—N1—C4—N2	−0.8 (2)	C11—C10—C15—O2	178.97 (16)
C1—N1—C4—N3	177.08 (13)	C11—C10—C15—C14	0.6 (3)
C1—C2—C3—N2	0.3 (3)	C11—C12—C13—C14	0.0 (3)
C3—N2—C4—N1	1.3 (2)	C12—C13—C14—O1	−179.37 (19)
C3—N2—C4—N3	−176.54 (14)	C12—C13—C14—C15	−1.4 (3)
C4—N1—C1—C2	−0.1 (2)	C13—C14—C15—O2	−177.46 (18)
C4—N2—C3—C2	−1.0 (2)	C13—C14—C15—C10	1.2 (3)
C4—N3—C5—C6	153.35 (14)	C14—O1—C16—O2	−16.4 (2)
C4—N3—C7—C8	−152.42 (14)	C15—O2—C16—O1	16.9 (2)
C5—N3—C4—N1	159.58 (14)	C15—C10—C11—C9	176.11 (14)
C5—N3—C4—N2	−22.4 (2)	C15—C10—C11—C12	−2.0 (2)
C5—N3—C7—C8	52.93 (18)	C16—O1—C14—C13	−172.2 (2)
C6—N4—C8—C7	60.01 (17)	C16—O1—C14—C15	9.7 (2)
C6—N4—C9—C11	−72.26 (17)	C16—O2—C15—C10	170.46 (18)
C7—N3—C4—N1	6.8 (2)	C16—O2—C15—C14	−11.0 (2)
C7—N3—C4—N2	−175.17 (13)		

### Hydrogen-bond geometry (Å, º)

Cg1 is the centroid of the pyrimidine ring and Cg2 is the centroid of the benzene ring.

**Table:**

*D*—H···*A*	*D*—H	H···*A*	*D*···*A*	*D*—H···*A*
C2—H2···*Cg*1^i^	0.93	2.83	3.6771 (17)	152
C9—H9*B*···*Cg*1^ii^	0.97	2.92	3.8090 (18)	152
C16—H16*A*···*Cg*2^iii^	0.97	2.80	3.689 (2)	153

Symmetry codes: (i) *x*, −*y*−1/2, *z*−1/2; (ii) *x*, *y*, *z*−1; (iii) *x*, −*y*+1/2, *z*−1/2.
